# Perpetual connectivity as digital labor: a conceptual reframing of the always-on work culture

**DOI:** 10.3389/fpubh.2026.1867925

**Published:** 2026-05-29

**Authors:** Nthabeleng Innocentia Mdhluli

**Affiliations:** Industrial and Organizational Psychology Department, University of South Africa (UNISA), Pretoria, South Africa

**Keywords:** attentional labor extraction, digital labor, HRM governance, informal digital labor, labor process theory, occupational health, perpetual connectivity, right to disconnect

## Abstract

**Orientation:**

Digital communication technologies have transformed the temporal and spatial organization of contemporary work, embedding continuous availability into organizational life. Despite considerable scholarly attention, the labor involved in maintaining perpetual digital availability remains under-theorized.

**Motivation for the study:**

HRM scholarship has predominantly treated connectivity as a contextual feature of digital work rather than as a labor process with direct occupational health implications. This framing obscures connectivity's function as a mechanism of attentional appropriation that warrants HR governance.

**Research purpose:**

This article reframes perpetual connectivity as a distinct form of digital labor. It argues that the always-on culture constitutes a site of value extraction and attentional appropriation that requires occupational health-grounded HRM theory and governance.

**Research design:**

A critical integrative review and theory synthesis was conducted, following Jaakkola (1). The synthesis integrates Labor Process Theory, Digital Sociology, and HRM scholarship.

**Findings:**

The article develops the Perpetual Connectivity Labor Model (PCLM), which specifies how organizational connectivity expectations generate informal digital labor through the mechanism of attentional labor extraction. Eight propositions guide empirical testing.

**Practical/managerial implications:**

The model offers HR practitioners a basis for governing digital responsiveness norms, recognizing informal digital labor within workload systems, implementing right-to-disconnect frameworks, and configuring HR analytics to monitor connectivity patterns.

**Contribution/value-Add:**

The article introduces attentional labor extraction as a novel explanatory mechanism, specifies the occupational health pathway from connectivity expectations to attentional depletion, and articulates a governance framework for digital work.

## 1 Introduction

Digital communication technologies have reshaped the temporal and spatial organization of work. Enterprise messaging platforms, collaboration tools, and mobile devices allow work to reach employees at virtually any moment, eroding the boundaries that once separated working from non-working time (2, 3). Continuous digital availability is now embedded in employment rather than functioning as a discretionary practice (4, 5). This transformation produces a recognizable occupational health hazard. Sustained after-hours connectivity is associated with psychological distress, burnout, and impaired recovery (6–8), and predicts diminished wellbeing and elevated turnover intention (9, 10). Connectivity also has documented benefits, including coordination across distributed teams, schedule autonomy for some workers, and inclusion across geographies and time zones (4, 10). The model developed here does not deny those gains. Its concern is more specific: the conditions under which the same infrastructure that enables flexibility produces systematic, organisationally generated exposure to harm.

Despite a substantial evidence base on outcomes, prevailing governance frameworks treat the resulting strain as individual stress rather than as workplace exposure (11, 12). The conceptual gap is most pronounced in human resource management (HRM) scholarship. Research has documented how digital tools generate technostress, blur work–nonwork boundaries, and intensify work demands (13–15), yet the labor process producing those outcomes remains under-specified. Connectivity is studied as a context in which work occurs rather than as work in itself (4, 16). The governance consequences are direct. If connectivity is only context, HR can offer wellness programmes and boundary management training but cannot intervene at the source of harm. If connectivity constitutes labor, HR becomes accountable for governing it through work design, policy, and infrastructure (17–20).

This article develops the Perpetual Connectivity Labor Model (PCLM), an occupational-health theory of perpetual connectivity with explicit HRM governance implications. The model traces a hazard–exposure–mechanism–outcome pathway. Organizational connectivity expectations constitute the hazard. Informal digital labor is the exposure pathway. Attentional labor extraction is the mechanism of harm. Attentional depletion, reduced recovery, and wellbeing impairment are the health outcomes, with turnover intention treated separately as a behavioral outcome.

Three questions structure the article. First, how should perpetual connectivity be conceptualized as a distinct form of digital labor within OHS and HRM theory? Second, through what mechanisms do organizational connectivity expectations generate informal digital labor, and how does that labor deplete employees' attentional resources? Third, under what conditions can HR systems govern these dynamics, and what testable propositions follow?

Although perpetual connectivity is a global phenomenon, its expression is shaped by local institutional conditions, and South Africa offers an analytically distinctive setting. Three features that elsewhere appear separately co-exist here: high structural inequality and unemployment, statutory working-time protections under the Basic Conditions of Employment Act, and active union engagement with digital working-time issues, including the right to disconnect (16, 17, 20, 21). These conditions sharpen specific propositions in the model rather than merely localizing it. Differential vulnerability is intensified where precarious employment is widespread, and workers have fewer structural resources to refuse availability demands (21, 22). Statutory floors and union mobilization operate as institutional moderators of policy embeddedness and cultural amplification, conditioning whether organizational connectivity governance is contestable or treated as private managerial prerogative (17, 20, 21, 23).

## 2 Methodology

The article is developed as a Hypothesis and Theory contribution following Jaakkola's (1) approach to theory synthesis, which generates conceptual contributions by integrating elements from distinct theoretical traditions. Theory synthesis is appropriate when no single tradition provides an adequate account of a phenomenon (1). Perpetual connectivity spans structural, normative, and governance dimensions that are addressed separately within Labor Process Theory, Digital Sociology, and HRM scholarship, and integrative theorization is therefore required.

### 2.1 Inclusion criteria

Sources were retained if they were peer-reviewed, in English, published 2000–2025, and engaged substantively with at least one of: connectivity or digital availability; digital labor and informal work; OHS-relevant outcomes (wellbeing, recovery, technostress, detachment); or HRM governance of digital work. Foundational older works from Labor Process Theory and Boundary Theory were retained where they remain canonical.

### 2.2 Exclusion criteria

Non-academic commentary, vendor reports lacking methodological transparency, and platform-labor studies without translatable insight for employed knowledge work were excluded unless cited specifically for boundary-condition discussion.

### 2.3 Search strategy

Scopus, Web of Science, and Google Scholar were searched using Boolean combinations across four blocks: connectivity (“*perpetual connectivity*,” “*always-on*,” “*after-hours connectivity*,” “*digital availability*”); labor (“*digital labor*,” “*informal digital labor*,” “*work intensification*”); OHS (“*technostress*,” “*psychological detachment*,” “*recovery*,” “*wellbeing*,” “*burnout*”); and governance (“*HR policy*,” “*right to disconnect*,” “*work design*”). Reference chaining and citation tracking supplemented the database searches.

### 2.4 Synthesis procedure

Development proceeded in four phases. The first mapped the three literature streams to identify their respective contributions and limits. The second placed these traditions in critical dialogue to surface complementarity and tension. The third specified the core constructs, including their boundary conditions and points of differentiation from related concepts. The fourth produced the PCLM and its propositions through iterative refinement. From an initial pool of approximately 520 records, 210 were assessed in full text, and 108 were retained on the basis of theoretical relevance, consistent with Jaakkola's (1) guidance for conceptual synthesis.

## 3 Literature review and theoretical foundation

### 3.1 Digital connectivity research

Research on digital work has documented the health consequences of constant connectivity. Tarafdar et al. (15) identify multiple stressors produced by technology use, including techno-overload, techno-invasion, and techno-complexity. Kreiner et al. (14) examine the cognitive and emotional work involved in negotiating work–nonwork boundaries within digitally saturated environments. Sonnentag and Bayer (8) establish that psychological detachment from work during off-job time is essential for recovery, and that connectivity systematically undermines this detachment. Ghai et al. (7) characterize the resulting configuration as a dilemma of connectivity in which organizations realize coordination benefits while employees absorb the psychological costs. These contributions are substantial, yet they share an analytical limitation. Connectivity is treated as a contextual variable that influences individual outcomes (11, 16). The labor process producing those outcomes receives comparatively little attention, as does the governance response required. The pattern reflects a broader tendency in occupational health research to focus on individual exposure and response while leaving the organizational sources of hazardous conditions under-theorized (24).

### 3.2 Engaging existing theoretical frameworks

Several established frameworks address aspects of perpetual connectivity. Each contributes to understanding, and each leaves a specific gap that the PCLM is designed to address.

Job Demands–Resources (JD-R) theory offers a strong account of how demands deplete energy and how resources buffer that depletion (25). Contemporary JD-R scholarship recognizes that demands are organisationally produced and addresses imbalance between demands and resources at the work-design level (10, 25). Its analytic center, however, remains the energetic and motivational response of the individual to demands taken as given. JD-R can explain why connectivity harms employees; it does less to specify why organizations sustain the conditions that cause harm and how those conditions might be governed at source.

Boundary theory examines how employees manage work–nonwork separation (14, 26). Recent work on boundary supplies, team-level boundary norms, and organizational boundary tactics has moved beyond a purely individual-coping account (27). The PCLM extends this trajectory by specifying the labor content of cross-boundary activity and the organizational process that generates it, rather than restricting analysis to the strategies through which employees absorb boundary pressure.

Work intensification research documents the increased pace and density of work, including under flexible arrangements (13, 28, 29). It addresses what occurs within working time. The PCLM addresses the qualitatively different problem of attentional extension into non-working time, where the unit of cost is fragmented attention rather than densified hours.

Role overload research links excessive task demands to stress and work–family conflict (30, 31). It treats overload as a quantity problem and does not theorize connectivity as a distinctive demand whose cost is structured around vigilance and anticipation rather than task volume.

Social exchange theory reveals a voluntary reciprocity for organizational support (32). It explains a portion of after-hours responsiveness, particularly where genuine reciprocity is present. It is less suited to compelled compliance, fear-based responsiveness, or impression management, where surface behaviors may resemble reciprocity while reflecting power asymmetry rather than discretionary exchange.

Strategic HRM frameworks recognize the importance of work design, employee voice, and policy (19, 33). They have not yet been applied systematically to connectivity as labor requiring governance (34, 35).

The pattern across these frameworks is consistent. Consequences are well documented; process is under-specified; and governance, where treated, sits at the level of individual support rather than organizational hazard control. The PCLM does not displace these frameworks. It draws on them and fills the integrative gap that they collectively produce.

### 3.3 Labor process theory and digital work

Labor Process Theory examines how work is controlled and how value is extracted from labor. Braverman (36) argued that employment contracts purchase the capacity to work rather than work itself, and that securing actual labor therefore requires ongoing managerial control. Fuchs and Sevignani (37) and Graham et al. (38) extend the analysis to digital contexts, demonstrating how digital availability enables organizations to extract value beyond formal working time. Klur and Nies (39) describe how digital technologies generate self-perpetuating dynamics of control, while Nies (40) introduces the notion of a politics of performance in which managerial strategies and worker involvement jointly define what counts as adequate engagement. Most Labor Process Theory scholarship on digital labor has examined platform workers (22, 38, 41), with comparatively limited engagement with employed knowledge workers (42). The tradition has also engaged only indirectly with HR governance (24). Its primary contribution to the PCLM is structural in that it explains why organizations possess systemic incentives to sustain connectivity expectations even where those expectations damage employees.

### 3.4 Digital sociology

Digital Sociology examines how connectivity expectations are interpreted and become normalized within professional life (43, 44). Where Labor Process Theory explains why organizations seek extended engagement, Digital Sociology illuminates how workers come to accept those expectations as constitutive of professional identity (45). Arantes and Vicars (45) show how the expectation of constant digital presence reshapes academic identity, transforming not only what academics do but how they understand themselves. Kaun (12) characterizes disconnection as a moral and political act rather than a purely technical one, carrying cultural meanings about productivity and commitment. Rice and Hagen (46) trace the generational normalization of perpetual contact across social life. Together, these studies show that connectivity expectations are sustained by cultural meaning, not solely by managerial pressure (47). Digital Sociology also attends to the design of digital tools. Read receipts, online status indicators, and response-time metrics are not neutral specifications. They render availability visible and generate social pressure to respond (3, 48). The PCLM accordingly treats digital infrastructure as an active driver of connectivity dynamics rather than a passive medium.

### 3.5 Integration

The PCLM integrates these traditions into a framework that is structural, normative, and governable. Labor Process Theory supplies the analysis of value extraction and control (36, 37). Digital Sociology supplies the account of norm formation and cultural meaning (12, 45). HRM and OHS scholarship supply the governance logic (19, 25). The integration is generative rather than additive. Structural pressures do not produce employee behavior mechanically; they operate through interpretive processes in which employees make sense of expectations and develop standards of conduct (47). Table 1 summarizes the contributions of each tradition.

**Table 1:** Theoretical traditions integrated in the PCLM.

Tradition	Core focus	Contribution to the PCLM	Limitation addressed
Labor process theory	Control and extraction	Specifies why organizations have structural incentives to sustain connectivity expectations and how digital availability extends value extraction (36, 37)	Extends analysis from platform workers to employed knowledge workers; introduces HR governance logic
Digital sociology	Norms and meaning	Specifies how connectivity expectations are internalized as constitutive of professional identity (12, 45)	Adds structural constraint and governance to interpretive accounts
Job demands–resources theory	Demands and resources	Links connectivity demands to well-being through energetic depletion (25)	Reframes connectivity as labor generating value, not only as a depleting demand
Boundary theory	Work–nonwork management	Specifies how employees negotiate work–nonwork separation (14, 26, 27)	Shifts focus from individual coping to organizational governance
Work intensification	Effort within working time	Documents accelerated work pace under flexible arrangements (13, 29)	Adds the temporal extension of labor into non-work time
Role overload	Excessive task demands	Links demand excess to stress and conflict (30, 31)	Theorizes connectivity as a distinct demand with attentional rather than purely quantitative structure

## 4 Conceptualizing perpetual connectivity as labor

### 4.1 Defining perpetual connectivity

Perpetual connectivity refers to the continuous expectation, explicit or implicit, that employees remain digitally available for work-related interaction beyond formal working hours (6, 27). It is distinct from work intensification, which concerns pace within working time (29), and from overtime, which involves formally extended hours (13). What characterizes perpetual connectivity is the ongoing potential for work intrusion (2): an attentional state of readiness that persists regardless of whether any specific demand is currently active. The distinction matters because connectivity can generate labor in the absence of active engagement. An employee monitoring messages during a family meal performs attentional work even when no message arrives (7, 49). A significant share of this work falls outside the temporal measures conventionally used to govern workload (4).

### 4.2 Informal digital labor

Informal digital labor (IDL) is the unbounded, fragmented, attentional, and affective activity through which employees maintain digital availability beyond formal working hours under organizational expectation, generating value for the organization while remaining unrecognized within formal workload systems (24, 37). The construct is restricted by the presence of organizational expectation. This restriction is critical to the model and addresses a common source of conceptual ambiguity in connectivity studies in which not all after-hours digital activity is considered labor in the relevant sense. Table 2 describes the typology used in this study.

**Table 2:** Distinguishing informal digital labor from related forms of after-hours availability.

Form of after-hours availability	Organizational expectation	Reward/ sanction	Compensation	Realistic capacity to opt out	Classification
Compulsory availability (formal on-call)	Yes	Yes (formal)	Yes (formal)	No	Formal labor; not IDL
Compensated overtime	Yes	Yes (formal)	Yes	Constrained	Formal labor; not IDL
Informal digital labor	Yes (often implicit)	Yes (informal)	No	Limited	IDL
Identity-driven self-expectation	Not organizational	None imposed	No	Yes	Personal practice; not IDL
Voluntary engagement (e.g., interest-driven)	No	None imposed	No	Yes	Personal practice; not IDL

In the context of a family meal, monitoring messages counts as labor in the IDL sense only when there is an organizational expectation, a reward or sanction is attached to responsiveness, and the realistic capacity to opt out is limited. When these conditions are not met, the behavior is more accurately classified as personal practice motivated by professional identity, work involvement, or boundary-integration preference. The distinction has implications for governance because organizational responsibility extends to the labor component of after-hours activity, not discretionary practice.

### 4.3 Four characteristics define IDL

*Unboundedness* denotes the absence of clear temporal limits. Whereas formal tasks have defined start and end points, IDL does not (50); it occurs during commutes, evenings, and weekends, occupying the interstices of daily life (2). Standard workload measures, which count hours and tasks, cannot capture it (4).

*Fragmentation* describes the micro-task structure of connectivity work. Brief, recurring actions like checking messages, monitoring channels, and acknowledging queries only take seconds, but the cumulative cognitive cost is significant because task switching imposes penalties that go beyond the immediate action (10, 15).

*Attentional demand* captures the vigilance required to maintain readiness. Even without active responding, employees monitor for incoming demands (49). Ghai et al. (7) demonstrate that this psychological availability mediates the relationship between connectivity and wellbeing. Vigilance is cognitively demanding even when it produces no observable output (8).

*Affective labor* is the emotional and relational work involved in maintaining responsiveness. It includes managing frustration at intrusions, sustaining a professional tone in after-hours interactions, and negotiating others' expectations of availability (51, 52). Following Hochschild (51), this affective dimension constitutes work even when it remains unrecognized as such.

### 4.4 Attentional labor extraction

Attentional labor extraction (ALE) is the organizational process by which connectivity expectations and the digital infrastructures that support them appropriate employee cognitive attention as a resource for organizational coordination, beyond the temporal and contractual scope of formal work (37, 48, 53). A point of conceptual hygiene is essential here. IDL and ALE are defined at different levels of analysis. IDL is a behavioral construct: what employees do to maintain availability. ALE is a relational and organizational process: how organizations structurally generate and capture attentional output through those behaviors. The behavior is the observable surface; the extraction process is the underlying organizational dynamic. By treating the two as distinct levels, the apparent circularity of utilizing attentional demand to define both the exposure and the mechanism is avoided. This is due to the notion that the exposure (IDL) operates at the level of employee activity, whereas the mechanism (ALE) operates at the level of organizational appropriation.

Three features distinguish ALE. It is continuous, operating across time rather than only during working hours (2). It is anticipatory, since readiness consumes attentional resources before any specific task arrives (49). It is appropriative, since sustained availability benefits the organization through accelerated coordination while employees bear the cost through depletion and reduced recovery capacity (8, 37). Operationally, ALE is observable when organizational expectation, infrastructure-enabled visibility of compliance, and behavioral evidence of sustained vigilance, fragmentation, or anticipatory monitoring co-occur. Its consequence (attentional depletion) is measurable through validated cognitive and recovery indicators. Within the model, ALE is the mechanism that links exposure to outcome. It explains how connectivity expectations generate attentional depletion (8, 49). Specifying this mechanism allows for intervention at the cause level rather than just the symptom level.

### 4.5 Construct differentiation

Attentional labor extraction differs from a family of related constructs in clearly specifiable ways. Table 3 illustrates these differences along the dimensions of level of analysis, core mechanism, antecedent locus, primary consequence, and the specific aspect that each construct fails to capture that ALE addresses.

**Table 3:** Differentiating attentional labor extraction from related constructs.

Construct	Level of analysis	Core mechanism	Antecedent locus	Primary consequence	Distinction from ALE
Technostress (15)	Individual experience	Strain from technology use	Technology features	Strain	ALE specifies the organizational process producing the conditions of strain; technostress is the felt experience
Workplace telepressure	Individual cognition	Preoccupation with response and rumination	Norms; technology	Rumination; impaired recovery	ALE is upstream organizational appropriation; telepressure is a downstream cognitive state
Availability pressure/ICT availability demands	Individual experience	Felt obligation to be available	Norms; technology	Strain; conflict	ALE specifies what is appropriated (attention) and how, beyond felt pressure
Work-related smartphone use after hours	Behavioral	Frequency of use	Norms; technology	Various	ALE is the organizational dynamic; smartphone use is one observable indicator of IDL
Psychological (non-) detachment (8)	Recovery state	Mental disengagement from work	Connectivity; demands	Recovery quality	ALE is upstream; detachment is the recovery state extraction undermines
Work-related rumination	Cognitive state	Repetitive thought about work	Demands; affect	Strain; impaired recovery	ALE is the organizational process; rumination is an individual cognitive symptom
Boundary supplies/boundary tactics (14, 26, 27)	Individual coping; team-level supplies	Strategies of separation	Boundary norms	Boundary management quality	Boundary work is the response to extraction; ALE specifies the organizational process generating the pressure
The ‘electronic leash'	Metaphor	Continuous tether	Technology	Constraint on autonomy	ALE is a theorized process specifying mechanism, level, and consequence; the leash metaphor lacks process specification
Constant connectivity to work (6)	Condition/state	Continuous availability	Technology; norms	Various	ALE specifies the labor content and extraction process internal to the condition
Work intensification (29)	Effort within working time	Pace and density of effort	Work design; flexibility	Strain	ALE captures temporal extension and attentional structure outside of working time
Cognitive load (53)	Individual experience	Working-memory demand	Task structure	Performance; learning	ALE is the organizational process producing the conditions; cognitive load is an individual property
Boundaryless work (26)	Structural condition	Absence of work–nonwork separation	Work design	Various	Boundaryless work is a structural condition; ALE is a process operating within that condition

Two implications emerge. First, ALE has explanatory value that the existing constructs do not collectively supply. It specifies a continuous, anticipatory, and organisationally produced process that generates attentional output appropriated by the organization. Second, the construct is empirically testable. It can be operationalized through the joint presence of organizational expectation, infrastructure-enabled visibility, and behavioral evidence of extracted attentional capacity (vigilance, fragmentation, anticipatory monitoring), and its consequence (attentional depletion) can be measured through validated cognitive and recovery indicators.

## 5 Perpetual connectivity labor model

### 5.1 Overview

The PCLM is a four-level model that corresponds to the standard occupational health hazard framework. The risk is associated with organizational connectivity expectations (Level 1). Informal digital labor is the exposure pathway (Level 2). Attentional labor extraction is the cause of harm (Level 2 process). The immediate cognitive-affective outcomes (Level 3) include attentional depletion and perceived workload expansion. Reduced recovery, wellbeing impairment, and turnover intention are the distal outcomes (Level 4), with reduced recovery operating as a mediator and turnover intention treated as a behavioral rather than health outcome.

**Figure 1:**
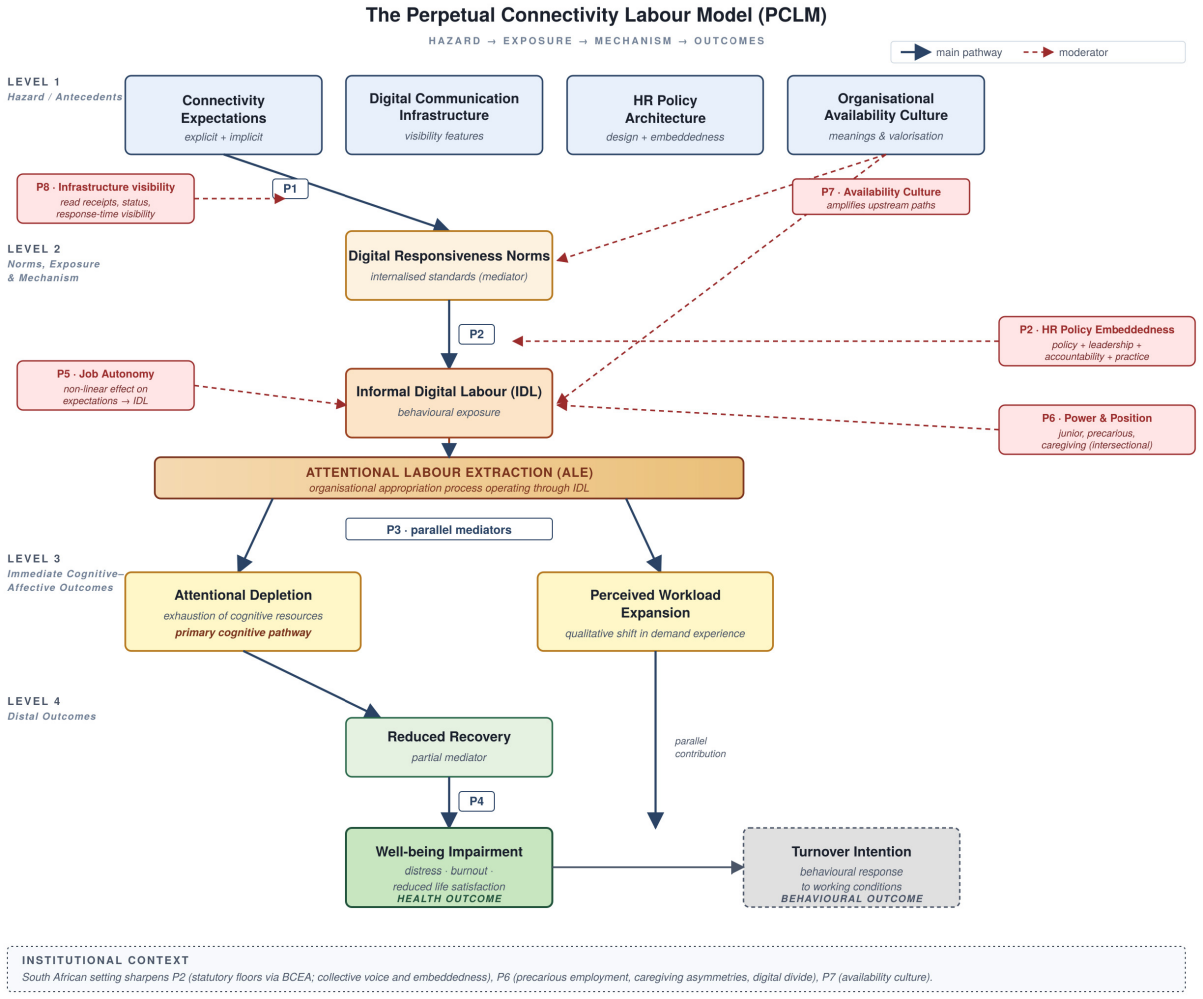
The perpetual connectivity labor model. Level 1 (Antecedents): connectivity expectations, digital communication infrastructure, HR policy architecture, and organizational availability culture. Level 2 (Mechanism): digital responsiveness norms (mediator), informal digital labor (exposure behavior), and attentional labor extraction (organizational process operating through both). Level 3 (Immediate outcomes): attentional depletion (primary) and perceived workload expansion (parallel). Level 4 (Distal outcomes): reduced recovery (mediator), wellbeing impairment (health outcome), and turnover intention (behavioral outcome). Moderators are mapped to specific pathways: infrastructure visibility features (P8) at the connectivity-expectations-to-norms pathway; HR policy embeddedness (P2) at the norms-to-IDL pathway; job autonomy (P5) at the connectivity-expectations-to-IDL pathway; power and position (P6) at the upstream pathways; availability culture (P7) at the upstream pathways only.

The model is presented in Figure 1. Each variable is assigned an explicit theoretical role such as antecedent, mediator, mechanism, moderator, or outcome. This consistency of role across models and propositions is critical to the model's testability.

Two roles in particular merit clarification, because they were under-specified in earlier formulations. HR policy architecture operates at two distinct levels. Its design status (whether policies exist at all and what they contain) is an antecedent at Level 1, because organizational design choices about policy frameworks shape the connectivity environment. Its effect on behavior, however, operates as a moderator of the norms-to-IDL pathway, mediated by embeddedness. The two roles are theorized separately to avoid confusion. Organizational availability culture similarly performs two roles. It is an antecedent of connectivity expectations because cultural valorisation of availability shapes what managers and peers signal as expected. It also functions as a moderator of upstream pathways because culture conditions how expectations are interpreted and translated into norms and behavior. Both roles are made clear in the figure and in the propositions.

### 5.2 Level 1: antecedents

Organizational connectivity expectations are explicit and implicit demands for digital availability outside regular working hours (4, 27). Explicit expectations are stated through policy, manager communication, or contractual arrangement. Implicit expectations are inferred from manager behavior, peer practice, and cultural signals (2, 47). An organization may publish no after-hours policy yet generate strong implicit expectations because managers respond at all hours, slow responses correlate with negative evaluation, or commitment is culturally equated with constant presence (2, 16). Implicit expectations are often more powerful than explicit ones because they are harder to identify and challenge (27). Digital communication infrastructure includes messaging platforms, mobile devices, remote-access tools, and collaboration software (48). They are not neutral channels. Read receipts, online status indicators, and push notifications make availability visible, generating surveillance effects and normative pressure to respond (3, 48). Infrastructure is therefore an antecedent in its own right, and platform configuration is a governance decision in which HR has a legitimate role (34, 35). HR policy architecture refers to the design quality and substantive content of organizational policies that govern connectivity. Its presence at Level 1 reflects the antecedent role of policy design. Its effect on behavior is moderated by embeddedness, theorized at Pathway 2. Organizational availability culture refers to the meanings the organization attaches to availability, dedication, and commitment (2, 12, 45, 47). Cultures that emphasize availability translate connectivity expectations into stronger normative pressure; cultures that protect non-working time soften that translation.

### 5.3 Level 2: norms, behavior, and the extraction process

Digital responsiveness norms are the internalized standards employees develop regarding appropriate availability: how quickly to respond, when to be online, what counts as acceptable delay (6, 52). Norms are not passively accepted. They are constructed through observation of colleagues, managerial feedback, and professional identity (45, 47), and they form simultaneously at individual, team, and organizational levels. Informal digital labor enacts these norms behaviorally, in the form specified in Section 4.2. It is also the surface through which attentional labor extraction operates: the same monitoring and responsiveness behaviors that maintain availability also constitute the appropriation of attentional capacity (12, 49). Attentional labor extraction is the organizational process that operates through the norm–behavior pairing. As specified in Section 4.3, it is positioned at a different level of analysis from IDL and is the explanatory mechanism linking exposure to harm.

### 5.4 Level 3 and 4: outcomes

Perceived workload expansion is the perception that demands have increased while boundaries have weakened. It does not necessarily reflect more hours worked (10), but rather a qualitative shift in how work is perceived as persistent, difficult to complete, and resistant to closure (15). The fragmentation and vigilance of IDL generate perceived expansion that may be disproportionate to any change in formal task load (7). Attentional depletion is defined as the exhaustion of cognitive resources caused by prolonged vigilance, task switching, and anticipatory monitoring (8, 49). It is a direct result of attentional labor extraction and the primary cognitive pathway that connects IDL to downstream health outcomes (8, 15). Attentional depletion and perceived workload expansion are parallel immediate outcomes of IDL, rather than sequential mediators. Perceived workload expansion operates through appraisal and demand-related strain, while attentional depletion operates through cognitive–physiological exhaustion and impaired recovery. Both contribute to the downstream outcomes, however, through partially distinct pathways.

Reduced recovery mediates the link between attentional depletion and wellbeing impairment. Recovery necessitates psychological detachment during off-work hours (8), and connectivity consistently undermines that detachment (52). The recovery deficit, rather than depletion alone, is what translates immediate cognitive cost into sustained wellbeing impairment. Wellbeing impairment includes psychological distress, burnout, and reduced life satisfaction (7, 11, 17). It is treated as the model's primary health outcome. Turnover intention is treated as a behavioral and organizational outcome rather than a health outcome (9). It is included in the model because it represents one of the most consequential downstream effects for organizations and because the evidence linking sustained connectivity to turnover intention is strong (9, 10). Its placement outside the OHS-outcome bracket is conceptual rather than empirical since it follows from the recognition that employee withdrawal is a behavioral response to working conditions, not a measure of health status.

### 5.5 Moderating conditions

Five moderating conditions are specified, with each mapped to a specific pathway rather than the model as a whole.

*Digital infrastructure visibility features* moderate the connectivity-expectations-to-responsiveness-norms pathway (P8). Features that render availability and response time visible to peers and managers strengthen the translation of expectation into norm by making compliance and noncompliance socially comparable.

*HR policy embeddedness* moderates the response-norms-to-IDL pathway (P2). Embeddedness is treated as a continuous variable, defined as the degree of alignment between formal policy, leadership modeling, accountability mechanisms, and cultural practice. High embeddedness dampens the translation of norms into IDL. Low embeddedness produces weak, inconsistent, or counterproductive effects, including the perception of organizational hypocrisy, displacement of responsibility onto employees, and increased cynicism toward HR interventions.

*Job autonomy* moderates the connectivity-expectations-to-IDL pathway in a non-linear manner (P5). At low expectation levels, autonomy reduces IDL by allowing flexibility in availability management (27). At high expectation levels, autonomy can increase IDL because the discretionary space becomes a stage on which visible responsiveness signals commitment (2).

*Power and position* moderate the strength of the upstream pathways (P6). Junior employees, those in precarious employment, and workers with significant caregiving responsibilities face greater exposure and lower realistic capacity to refuse demands (21, 22, 40).

*Organizational availability culture* moderates the upstream pathways specifically (P7): expectations-to-norms and norms-to-IDL. It does not necessarily strengthen the downstream pathway from depletion to wellbeing impairment, which is governed largely by physiological and recovery dynamics that are not culturally amplified in the same way.

### 5.6 Causal pathways

The level structure creates five pathways. Pathway 1 connects connectivity expectations to responsiveness norms through observation, sense-making, and social learning (45, 47), strengthened by infrastructure visibility features. Pathway 2 connects norms to IDL as employees enact internalized standards (6), moderated by HR policy embeddedness. Pathway 3 connects IDL to perceived workload expansion through fragmentation and cumulative cognitive demand (15). Pathway 4 connects IDL to attentional depletion through attentional labor extraction (8, 49). Pathway 5 connects the immediate outcomes (depletion in particular) through reduced recovery to wellbeing impairment, with parallel contributions from perceived workload expansion (7–9). Together, these levels and pathways describe a single causal process in which connectivity expectations generate norms, norms generate informal digital labor, that labor is the surface of attentional labor extraction, and extraction depletes the cognitive resources required for recovery and wellbeing. The eight propositions in the following section divide this process into testable claims.

## 6 Propositions

The PCLM produces eight empirically testable propositions. Each proposition states one theoretical claim, with main effects and moderating effects clearly separated to support empirical testing and to avoid the kind of compound proposition that conflates different theoretical roles.

### 6.1 Proposition 1

Organizational connectivity expectations are positively associated with digital responsiveness norms.

### 6.2 Proposition 2

The degree of HR policy embeddedness, defined as the alignment between formal policy, leadership modeling, accountability mechanisms, and cultural practice, negatively moderates the relationship between digital responsiveness norms and informal digital labor. Low embeddedness has weak, inconsistent, or counterproductive consequences, including perceptions of organizational hypocrisy and increased self-monitoring.

### 6.3 Proposition 3

Informal digital labor is positively associated with attentional depletion through attentional labor extraction. Informal digital labor is also positively associated with perceived workload expansion. Attentional depletion and perceived workload expansion operate as parallel cognitive–affective consequences of informal digital labor.

### 6.4 Proposition 4

Attentional depletion is negatively associated with employee wellbeing, with reduced recovery serving as a partial mediator of this relationship.

### 6.5 Proposition 5

Job autonomy moderates the relationship between connectivity expectations and informal digital labor in a non-linear manner. At low expectation levels, autonomy reduces informal digital labor. At high expectation levels, autonomy increases informal digital labor by widening the discretionary space within which visible responsiveness becomes a signal of commitment.

### 6.6 Proposition 6

Connectivity expectations exert a stronger effect on informal digital labor and downstream outcomes among employees in junior positions, precarious employment, or with significant caregiving responsibilities. In labor markets characterized by high unemployment, weak enforcement of working-time protections, or substantial informality (South Africa is a salient example), this differential is intensified by reduced realistic capacity to refuse availability demands.

### 6.7 Proposition 7

Organizational availability culture amplifies the upstream pathways of the model, specifically the effect of connectivity expectations on responsiveness norms and the effect of responsiveness norms on informal digital labor. It does not necessarily strengthen the downstream pathway from attentional depletion to wellbeing impairment, which is governed primarily by recovery and physiological dynamics.

### 6.8 Proposition 8

Digital communication infrastructures with high-visibility features, such as read receipts, online status indicators, and response-time visibility, strengthen the relationship between connectivity expectations and digital responsiveness norms by rendering compliance and noncompliance socially comparable.

## 7 Theoretical contribution

The article makes five contributions to OHS, HRM, and digital work scholarship.

Perpetual connectivity is reframed as an occupational health hazard. Existing research examines effects of connectivity on health while accepting connectivity as a given context (11, 15, 16). The PCLM repositions connectivity as a manageable occupational hazard with a specifiable causal process from exposure to outcome. The shift moves governance accountability from individual coping to organizational responsibility (19) and broadens HR's mandate from symptom management to hazard governance.

Specifying attentional labor extraction as a theoretical mechanism. ALE describes how organizations systematically appropriate cognitive attention through connectivity demands, offering a theoretical account of organizational generation rather than individual reaction. Unlike technostress, it specifies process rather than experiential outcome (15). Unlike work intensification, it operates across temporal boundaries rather than within them (29). Unlike cognitive load, it is relational and organizational rather than individual and experiential (53). Defining it at a level of analysis distinct from IDL avoids the circularity that arises when exposure and mechanism are described at the same level. By naming the mechanism, the model enables empirical investigation and cause-specific intervention.

Identifying informal digital labor as a theoretically grounded construct. The four characteristics of unboundedness, fragmentation, attentional demand, and affective labor, together with the boundary conditions distinguishing IDL from other forms of after-hours availability (Table 2), produce a construct with clear empirical referents. The specification facilitates measurement development and distinguishes connectivity work qualitatively from formal task requirements (15, 37, 51).

Extending Labor Process Theory to Employed Knowledge Workers. Labor Process Theory has produced detailed analyses of platform workers (22, 38, 41), with relatively little engagement with the normative and cultural mechanisms that subject professional knowledge workers to extended control. The PCLM extends LPT into this territory while incorporating HRM governance logic (19), linking structural accounts of digital capitalism to individual occupational health experience.

Positioning HR systems as occupational health governance tools. The embeddedness specification of HR policy (P2) defines the conditions under which HR intervention can effectively moderate the norms-to-labor pathway (20, 35). This moves beyond presence-or-absence approaches to policy evaluation and identifies the implementation conditions, namely leadership modeling, accountability, and cultural alignment, that determine policy efficacy (19).

## 8 Discussion

### 8.1 What the PCLM adds

Three explanatory advances distinguish the PCLM from neighboring frameworks. JD-R theory accounts for the depletion mechanism (25) but does not explain why organizations sustain the conditions that cause depletion. ALE addresses this question by demonstrating that organizations derive value from continuous availability through accelerated coordination and extended operational capacity (37, 50). Depletion is therefore an externality of productive activity rather than an unintended consequence (24). The direct implication for governance is that interventions that do not address the organizational interests served by connectivity are unlikely to produce durable change.

Boundary theory examines how employees manage work–nonwork separation under connectivity conditions (14, 26, 27), and contemporary work increasingly attends to organizational boundary supplies and team-level norms. The PCLM extends this trajectory by specifying the labor content of cross-boundary activity and the organizational process that generates it. The reorientation matters because it shifts a portion of the governance burden from individual coping skills, which are unevenly distributed across the workforce, to organizational expectations, which can be addressed through policy, leadership, and structural design (18, 20).

Labor Process Theory offers a structural analysis of extraction in platform labor (38, 41), where extraction operates largely through algorithmic direction. Knowledge workers experience extraction through cultural expectations and professional identity (2, 45). The PCLM adapts the theory to that empirical setting, with implications for governance: in these contexts, intervention must address norms and meaning, not just contractual obligations (12).

### 8.2 The dual nature of connectivity

The model does not present a one-sided view of digital connectivity. Connectivity facilitates coordination, schedule autonomy for some employees, and inclusion in distributed teams (4, 10). The PCLM addresses the circumstances in which the same infrastructure becomes hazardous. Three conditions are particularly important. The initial step is the configuration of infrastructure. High-visibility features, such as read receipts and status indicators, convert availability into a measurable performance signal (3, 48). The second factor is the embeddedness of policy: connectivity governance is at best inert and at worst counterproductive when formal policy is symbolic rather than embedded (35). The third is the cultural value of availability, where commitment is equated with constant presence, expectations translate strongly into norms and IDL (2, 45). The model identifies these conditions, allowing organizations to maintain the coordination and inclusion benefits of connectivity while constraining the hazard pathway.

### 8.3 The South African Context

South Africa exemplifies a setting in which the model's institutional moderators are pronounced and analytically visible.

High unemployment and substantial informal employment intensify the differential vulnerability described in P6 (21, 22). Workers in precarious arrangements have a sharply reduced realistic capacity to refuse availability demands. The asymmetry compounds existing inequalities along lines of class, race, and gender, especially where caregiving responsibilities are unevenly distributed (27).

The Basic Conditions of Employment Act establishes working-time protections that, where enforced, set a regulatory floor on the operating range of Level 1 connectivity expectations (17). Enforcement is uneven, particularly for informal and platform-mediated work (21), but the legal architecture provides a leverage point that organizations and unions can use to constrain the implicit expectations that drive IDL.

South African trade unions have engaged with the right to disconnect as a continuation of longstanding struggles over working time and decent work (20, 21, 23). Active union engagement is a structural condition that can shift HR policy embeddedness from symbolic toward embedded by introducing accountability and dialogue, directly engaging the moderator at the center of P2.

Inequality in access to and quality of digital infrastructure means that the population subject to extraction is itself stratified (16, 54). Middle-class professional workers experience identity-driven and infrastructure-mediated extraction; lower-skill workers in precarious employment are more likely to face compulsory availability with weaker access to formal protections and collective voice. The model's propositions therefore operate differently across these strata, and analysis of South African connectivity dynamics must attend to both segments.

These features are not typical of the region. They are systematic conditions that sharpen the model's propositions and that other middle-income economies share in varying combinations. Treating South Africa as a substantive analytical site rather than a passing illustration contributes to a more honest account of how connectivity governance operates beyond high-income, fully formal labor markets.

### 8.4 Occupational health governance implications

Current OHS governance approaches are structurally insufficient for connectivity because they focus on downstream effects rather than upstream causes (11, 18). Wellness programmes that help employees manage connectivity-related stress do not address the demands that produce the stress. Boundary management training does not change the expectations that drive the need for boundary management. Such interventions are inherently limited. Four upstream shifts are required. Workload assessment must account for connectivity demands; traditional metrics that count hours and tasks consistently underestimate the attentional burden of IDL (4). Work design must address connectivity expectations directly, incorporating protected attentional time and asynchronous collaboration into team processes (33). Performance management must focus on outcomes rather than availability, removing the implicit rewards for constant responsiveness that drive extraction (2). Digital infrastructure must be treated as an OHS governance domain, with platform configuration, notification design, and visibility features assessed for their measurable health impacts (48).

### 8.5 Research agenda

Numerous research streams are generated by the model. Measurement development must produce validated instruments for the core constructs, particularly attentional labor extraction and informal digital labor, with clear operational distinction from the related constructs in Table 3. Causal process testing using longitudinal and experimental designs should investigate the parallel-mediator structure of attentional depletion and perceived workload expansion (P3), the mediating role of reduced recovery (P4), and the moderating effects (P5–P8). HR governance research should compare organizations across the embeddedness continuum to test the moderating effect specified in P2. HR analytics research should develop ethical frameworks for monitoring connectivity patterns and assessing governance interventions, with attention to the privacy and surveillance risks that connectivity-related analytics themselves can generate (34). Comparative and contextual research should examine how institutional conditions shape connectivity dynamics. South Africa offers a particularly informative case (16, 21), but cross-national comparison across high- and middle-income labor markets is needed to specify the model's scope conditions. Longitudinal health research should document the cumulative health consequences of sustained extraction across career trajectories.

## 9 Practical implications

The PCLM provides HR practitioners with a governance framework based around five high-leverage interventions, each addressing a different aspect of the model.

Regulate connectivity expectations at source. HR must identify and challenge both explicit and implicit expectations regarding after-hours availability. Policies should define acceptable contact, distinguish urgent from routine communication, and be enforced through leadership modeling and accountability rather than published documents alone (2, 20). Worker representatives should be involved in policy development to ensure legitimacy and operational realism, particularly in unionized contexts (21).

Redesign work to reduce attentional demand. Job analysis should treat connectivity expectations as a workload component. Decision rights should be clarified to reduce after-hours consultation, and asynchronous collaboration should be integrated into team processes (33). Roles with protected attentional time and clear availability expectations produce less IDL than roles with ambiguous expectations.

Align performance management with sustainable performance. Performance criteria that implicitly reward responsiveness encourage extraction even where formal policy discourages it (2). Removing availability signals from evaluation frameworks, and recognizing sustainable performance, are necessary cultural-change levers (47).

Manage digital infrastructure as an OHS domain. Platform configuration shapes the attentional environment of work (3, 48). Notification design, visibility features, and the availability of do-not-disturb functionality have direct health consequences. HR should develop the capacity to evaluate these options and engage technology governance accordingly (34).

Address the differential vulnerability directly. Junior workers, those in precarious employment, and workers with significant caregiving responsibilities face heightened exposure and have fewer resources for resistance (21, 40). Governance interventions calibrated to these groups, rather than uniform programmes designed for the least vulnerable, are more equitable and more effective (17).

## 10 Limitations

The PCLM is conceptual and requires empirical testing to determine its validity and scope. Five limitations are particularly noteworthy. The model is primarily intended for employed knowledge workers and may require adaptation for other settings. Attentional dynamics central to the PCLM may operate differently in physical work, service work, or platform labor, where tasks are more tightly scripted or algorithmically directed (38, 55). Comparative research is required to specify the model's scope conditions. The differential vulnerability proposition (P6) identifies power and position as moderators but does not fully theorize how gender, race, and caregiving status interact with connectivity exposure. Emerging evidence suggests that women with caregiving responsibilities face additional and qualitatively distinct pressures (27). These intersections warrant further theoretical and empirical investigation. The South African contextualization is institutionally grounded but is itself a starting point. The propositions concerning labor protections and union engagement as moderating conditions require empirical testing in South African and other middle-income contexts. Institutional features of the South African labor market, including patterns of informal employment, public-sector employment relations, and the distinctive history of collective bargaining, may shape connectivity dynamics in ways the current formulation does not yet fully capture (17, 54). Validated measurement instruments for attentional labor extraction do not yet exist, and instrument development is therefore a precondition for large-scale quantitative testing (15, 53). The model's causal pathways imply temporal precedence that requires longitudinal or experimental verification rather than cross-sectional inference.

## 11 Conclusion

Perpetual connectivity is an occupational health hazard that organizations produce and that HR systems are accountable for governing. The Perpetual Connectivity Labor Model provides a framework for that governance. Connectivity expectations generate informal digital labor. Informal digital labor is the surface of attentional labor extraction. Extraction depletes the cognitive resources required for sustained performance and psychological recovery (8, 49). Wellbeing impairment is, on this account, a predictable consequence rather than an unintended one. The model's central contribution is explanatory clarity. Attentional labor extraction specifies how connectivity becomes a labor process and how that process produces health outcomes, in ways that technostress, work intensification, and cognitive load do not (15, 29, 53). It identifies the structural incentives that perpetuate the hazard, defines the conditions under which infrastructure and policy moderate the pathway, and directs governance to the source rather than the symptom. The challenge is both empirical and practical. The eight propositions provide a research agenda for testing, refining, and extending the model. The five governance interventions offer a framework for HR practitioners working in organizations where the always-on culture has become default rather than considered choice. Both rest on the same recognition that how organizations manage connectivity is an occupational health decision, and the costs of poor management are borne primarily by employees.
